# A “Wear and Tear” Hypothesis to Explain Sudden Infant Death Syndrome

**DOI:** 10.3389/fneur.2016.00180

**Published:** 2016-10-28

**Authors:** Eran Elhaik

**Affiliations:** ^1^Department of Animal and Plant Sciences, University of Sheffield, Sheffield, UK

**Keywords:** sudden infant death syndrome, allostatic load, neonatal circumcision, trauma, pain, stress

## Abstract

Sudden infant death syndrome (SIDS) is the leading cause of death among USA infants under 1 year of age accounting for ~2,700 deaths per year. Although formally SIDS dates back at least 2,000 years and was even mentioned in the Hebrew Bible (Kings 3:19), its etiology remains unexplained prompting the CDC to initiate a sudden unexpected infant death case registry in 2010. Due to their total dependence, the ability of the infant to allostatically regulate stressors and stress responses shaped by genetic and environmental factors is severely constrained. We propose that SIDS is the result of cumulative painful, stressful, or traumatic exposures that begin *in utero* and tax neonatal regulatory systems incompatible with allostasis. We also identify several putative biochemical mechanisms involved in SIDS. We argue that the important characteristics of SIDS, namely male predominance (60:40), the significantly different SIDS rate among USA Hispanics (80% lower) compared to whites, 50% of cases occurring between 7.6 and 17.6 weeks after birth with only 10% after 24.7 weeks, and seasonal variation with most cases occurring during winter, are all associated with common environmental stressors, such as neonatal circumcision and seasonal illnesses. We predict that neonatal circumcision is associated with hypersensitivity to pain and decreased heart rate variability, which increase the risk for SIDS. We also predict that neonatal male circumcision will account for the SIDS gender bias and that groups that practice high male circumcision rates, such as USA whites, will have higher SIDS rates compared to groups with lower circumcision rates. SIDS rates will also be higher in USA states where Medicaid covers circumcision and lower among people that do not practice neonatal circumcision and/or cannot afford to pay for circumcision. We last predict that winter-born premature infants who are circumcised will be at higher risk of SIDS compared to infants who experienced fewer nociceptive exposures. All these predictions are testable experimentally using animal models or cohort studies in humans. Our hypothesis provides new insights into novel risk factors for SIDS that can reduce its risk by modifying current infant care practices to reduce nociceptive exposures.

## Background

### The Etiology of Sudden Infant Death Syndrome

Sudden infant death syndrome (SIDS) (9ICD 798.0; 10ICD R95), “crib death,” or “cot death” was first coined in 1953 and by 2004 was defined as: “the sudden unexpected death of an infant under 1 year of age, with onset of the fatal episode apparently occurring during sleep, that remains unexplained after a thorough investigation, including performance of a complete autopsy and review of the circumstances of death and the clinical history” (1). SIDS classification spurred the development of diverse approaches to determine the cause of death in order to exclude deaths due to accidental or non-accidental injuries, suffocation and strangulation, or medical causes (2, 3). Despite the publication of ~11,000 SIDS-related articles (4), of which over 100 SIDS studies appearing in *Medical Hypotheses*, biomarkers are still unavailable (5) and SIDS remains the leading cause of death for infants between 1 month and 1 year in western countries (accounting for ~2,700 deaths per year in 2010 in USA) (6). In developed countries, the 2005 SIDS rates (birth to 1 year) ranged from 0.16 (Japan) to 0.54 (United States) per 1000 live births (7). Using non-Hispanic whites as a reference point (1), SIDS rates are 1.41 per 1000 live births in non-Hispanic blacks and 0.46 in Hispanics (8). In the absence of a proven intervention even if prospective identification was feasible, SIDS is one of the most frequent worries for parents.

Although prone sleeping campaigns, such as “Back to Sleep” reduced SIDS and postneonatal mortality rates in the 1990s (9), their effect was uneven in different countries (10). Subsequent to 1999, the plateau, or slower decline in SIDS rates, has been associated with a diagnostic shift from classification of SIDS deaths (11, 12) to a concurrent increase in rates of other categories of sleep associated sudden and unexpected infant deaths attributed to accidental suffocation and strangulation in bed or “unknown” causes (13), questioning the magnitude of the actual decrease in SIDS deaths (14, 15). While no confirmed SIDS biomarkers exist [e.g., Ref. (16, 17)] nor it is possible to differentiate accidental asphyxia from SIDS (18), a number of modifiable risk factors have been associated with SIDS, including prone sleep position, parental smoking during and after pregnancy, alcohol consumption by caregivers, overheating, preterm infants, infant head covering by soft bedding, bed sharing, and upper respiratory tract infection (19–21). Decreasing in these risk factors was effective in reducing the mortality rates over the past two decades (22), though not necessarily SIDS, which remains distinct from known mortalities and its main characteristics – male predominance (60:40 male:female USA ratio), significantly lower SIDS rates in USA Hispanics compared with whites, infants aged 2–4 months being at greatest risk of SIDS with most SIDS-related deaths occurring by 6 months, and seasonal variation with most cases occurring during winter (8, 23) – remain largely unexplained. Here, we propose an allostasis-based model to explain SIDS and argue that it explains SIDS’s main characteristics (Table 1).

**Table 1:** **The main characteristic of SIDS explained by the allostatic load hypothesis**.

SIDS characteristic	Allostatic load hypothesis
Male predominance (60:40)	Females are more resilient to nociceptive stimuli than males, which reduces their allostasis compared to males. Male circumcision further increases male allostasis.
SIDS rate in the USA vary between Hispanics and whites	Circumcision rates among Hispanics are much lower than in non-Hispanic white.
Mortality peaks between 2 and 4 months	Infants lose the protection of maternally acquired antibodies at 2–4 months of age. The wound healing procedure from circumcision may increase susceptibility to infection peaks during the same period. Preterm infants have also decreased heart rate variability at that period (corrected age).
Seasonal variation with most cases occurring during winter	Waning of maternal antibody levels and/or low levels of acquired immunity followed by recent infection and inflammation during a developmental period in the infant increase the allostatic load.

### The Allostasis Model

The allostasis model assigns a central role to the brain as the organ of stress and adaptation in enabling efficient regulation of the internal milieu (24). Facing low- or high-level toxic and pathologic stressors, the brain attempts to adapt via neuroendocrine and autonomic signals and through the synaptic plasticity facilitated by multiple epigenetic mechanisms during early development (25–27). The cumulative effect of stressors on the brain and body for either potentially protective or pathologic responses is termed *allostatic load* or *overload*, respectively (27). An allostatic overload occurs when allostatic responses that allow neurophysiologic stress systems to function at critical periods of the developing nervous system are maladaptive for future environmental stressors. The physiological cost of increased allostatic load has also been dubbed a “wear and tear” process (28) and encompasses both the prenatal and postnatal periods (29).

**Figure 1:**
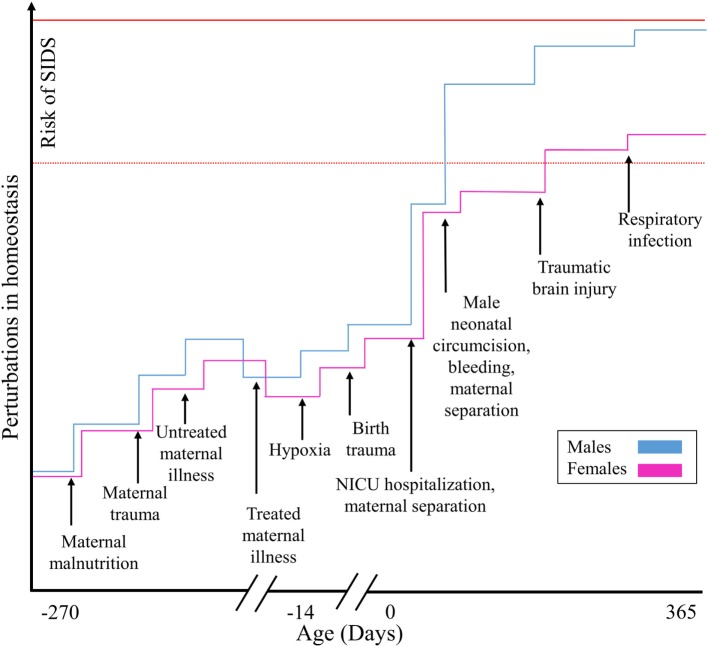
**Illustrating how SIDS is explained by the allostatic load model for males and females**. Cumulative stressful, painful, or traumatic stimuli contribute additively toward an increased risk of SIDS.

**Figure 2:**
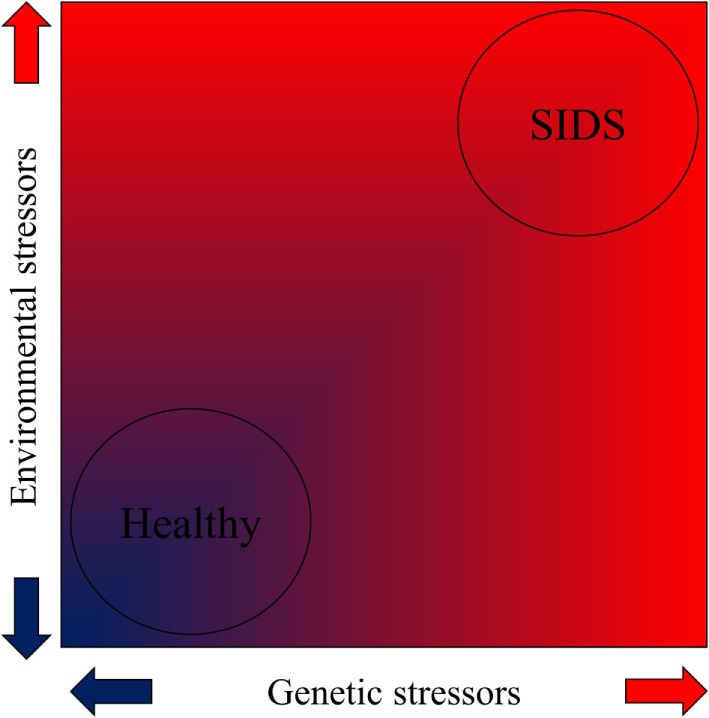
**The contribution of genetic and environmental stressors toward SIDS according to the allostatic load model as demonstrated by a heat map (red indicates high stress)**.

We postulate that while low-level stress can stimulate adaptation, prolonged and repetitive iatrogenic stressful, painful, or traumatic experiences during prenatal, perinatal, neonatal, and postneonatal development constitute allostatic overload and are risk factors for SIDS. Due to their total dependence, the infant’s ability to allostatically regulate exposure to stressors is severely constrained (30), which increases their vulnerability to disease and premature death (26) (Figures 1 and 2). Due to their difficulties in maintaining homeostasis and inability to escape/avoid iatrogenic or non-medically nociceptive exposure, infants are vulnerable to toxic stress with preterm infants being the most vulnerable (31).

### Stress Experienced *in utero*

Infants begin to experience various stresses *in utero* due to maternal smoking or drug abuse (19), caffeine consumption (32), maternal inflammation, and traumatic injuries that account toward allostatic load. Interestingly, several authors have shown sex-dependent changes in pain responsiveness at maturity in animals having experienced repeated neonatal pain *in utero* and infancy. For example, Mueller and Bale (33) showed that stress experienced early in pregnancy created maladaptive behavioral stress responsivity, anhedonia, and an increased sensitivity to selective serotonin reuptake inhibitor treatment. Provided the lower expression of DNMT1, the enzyme responsible for methylation maintenance in male placentas compared to females, the authors reasoned that females are able to circumvent the effects of stress by strengthening the maintenance of methylation during perturbations. Page et al. (34) reported a higher mechanical sensitivity in male rats in response to neonatal-paw-needle-stick, whereas females showed increased sensitivity to inflammatory stimuli. Such female “protective effects” may be attributed to hypothalamic–pituitary–adrenal (HPA) axis hormones known to be elevated in females both at baseline and in response to stress (35). Indeed, Paterson et al. (36) reported that SIDS cases had a significantly larger deficiency in serotonin 5-HT_1A_ receptors compared with controls [μ = 53.9 (σ = 19.8)] and that male SIDS cases had significantly lower receptor counts [16.2 (4.8)] compared with female SIDS cases [29.6 (16.5)].

### Preterm Births

It is known that the postnatal age for SIDS and other deaths of unknown causes decreases as gestational age (GA) at birth progresses. What remains unknown is why preterm infants remain at increased risk for sudden infant death despite the dramatic drop in rates since the 1990s and whether the increased risk across all categories of sudden infant death suggest a common mechanism (37). We speculate that preterm infants are at an increased risk due to their immature organ systems and the multiple stressors they experience during hospitalization and later on.

Preterm infants (24–32 weeks), physiologically unprepared for the stress outside the protective intrauterine environment, are hospitalized for lengthy periods where they are exposed to multiple stressors, including extended exposure to light, noise, acute and chronic illness, maternal separation, invasive procedures, handling, multiple medications, endotracheal intubation, repeated blood tests, insertion of peripheral lines, and surgery (38, 39). Preterm infants who experienced at least 40 days of intensive care have increased brain neuronal responses to noxious stimuli compared to healthy newborns at the same age (40). The cumulative effect of these early repetitive pain/adverse experiences on perinatal brain plasticity contributes to the observed neurodevelopmental and behavioral abnormalities associated with early life stress and allostasis (40–42).

Preterm infants are also at higher risk of hypoxia than term infants. Following hypoxia, infants maintain adequate tissue oxygenation through perfusion by prompting an increase in heart rate to maintain cardiac output. The heart is relatively insensitive to hypoxia in infancy, as opposed to hypercapnia, which rises rapidly during asphyxia and leads to adrenal and catecholamine release that increases heart rate and blood pressure (43). Infants who succumb to life-threatening cyanosis/asphyxia have been observed to breathe vigorously/gasp but without an increase in heart rate, suggesting an abnormal/impaired tachycardia response to hypercapnia despite ventilatory efforts (43). An impaired baroreflex sensitivity can result in a failure to control perfusion pressure. Very preterm infants have impaired and delayed maturation of baroreflex sensitivity (44) and decreased heart rate variability at peak SIDS age of 2–3 months (corrected age) consistent with delayed maturation of parasympathetic innervation regardless of sleeping position (45). Cerebral autoregulation is also impaired in preterm infants with decreased cerebral oxygenation in the prone position and increased variability in cerebral oxygenation with head up tilt (orthostatic challenge) indicating greater risk of cerebral hypoxia and immature cerebrovascular control in preterm infants over the first 6 months (46).

### Non-Urgent Pediatric Surgeries

Concerns that pediatric surgeries requiring general anesthesia are toxic stressors that elicit long-term deficits in cognitive and learning behavior have been the subject of recent reviews [e.g., Ref. (47, 48)]. The 2014 Food and Drug Administration (FDA) Science Board reviewing anesthetic neurotoxicity stated that the data “are sufficient to conclude that adverse effects noted in juvenile animals are reasonably expected to occur in developing humans” (49). The FDA Board recommended avoiding non-urgent surgical procedures in children younger than 3 years of age (48). In addition to general anesthesia, the risk of local anesthetic neurotoxicity, due to lack of adequate studies of safety and effectiveness in the developing infant, has recently been highlighted (50).

The surgery itself may elicit neural injury in neonates (51). The significantly increased risk of death or neurodevelopmental impairment in very low birth weight newborns following major or minor surgery that does not require a general anesthetic (52) is consistent with nociceptive exposure, pain, and overall neurotoxic risk (47, 53, 54). The American Academy of Pediatrics (AAP) and the Canadian Pediatric Society (CPS) have recognized the neurotoxic risk of pain in their joint policy statement urging the avoidance, prevention, and possible elimination of pain even during routine minor procedures to protect the developing brain (55). Therefore, the safest course of action is avoidance of non-medical and non-urgent surgeries, as recommended by the FDA Science Board, as well as avoidance of iatrogenic procedural pain, as recommended by AAP–CPS, to protect the developing brain in infants. While pediatric surgeries are typically rare and cannot account for the high rates of SIDS, particularly if the cause of death is ascribed to the surgery, we suspect that a specific type of voluntary painful surgery concealed under the cloak of routine hospital practices account for the high volume of SIDS rates.

### Neonatal Circumcision

Circumcision is one of the most common elective surgical procedures in the world and is performed primarily on males (56). Female circumcision is practiced in nearly 30 African countries, some Southeast Asian and Middle Eastern countries, and in immigrant communities in Europe and North America (57). Despite its relevancy, female neonatal circumcision will not be discussed here since in most western countries it is illegal and thereby under-reported and we lack SIDS data for the remaining countries. In North America, ~1.2 million male infants are circumcised every year (58) often within the first 2 days of life (59). Although not requiring general anesthesia, circumcision is an intensively painful procedure requiring adequate analgesia (60). Circumcision is associated with intraoperative and postoperative risks, including bleeding, shock, sepsis, circulatory shock, and hemorrhage (61–63) that can result in death (63, 64).

Infant deaths following religious neonatal circumcision have been known for at least two millennia (65). Talmud (the central text of Rabbinic Judaism) sages ruled in the first centuries A.D. that mothers with two children who have died following the surgery should receive an exemption from circumcising their infants. During the nineteenth century, developments in medical knowledge on one hand and the rise of Jewish “Enlightenment” on the other hand, brought many Jews to reject the authority of the Talmud and with that the practice of circumcision. A new wave of accusations toward Jewish circumcisers (mohels) and rabbis of infant deaths following circumcision soon appeared and prompted community leaders to appeal to the governing authorities to forbid this practice – efforts that were countered by rabbis’ threats to ban the admission of uncircumcised Jewish children from Jewish schools. The fierce arguments about the necessity of the procedure last to this day and led many Jews to opt their infants out of the procedure, including Theodor Herzl, one of the fathers of modern political Zionism (66). In the UK, Gairdner (67) estimated an annual rate of 16 per 100,000 circumcision-associated deaths for boys under 1-year old in a study that influenced the British government to exclude circumcision coverage from the National Health Service. Remarkably, the SIDS rates in the UK (0.38 per 1000) are much lower than in the USA (0.55 per 1000) (10) where most male infants are circumcised (58). Moreover, most of the deaths in the USA occur in non-Hispanic blacks (83% higher death rate compared with non-Hispanic whites). SIDS rates were 44% lower for Hispanics compared with non-Hispanic whites (68). Interestingly the circumcision rates among Hispanics are about half that of the two other groups (69).

Circumcision contributes to the rise in allostatic load and increased risk for SIDS through multiple conduits. Circumcision produces crush and incisional injuries during amputation, resulting in damage to normal prepuce tissue, the associated nerves, and blood vessels. Wound healing manifested by hyperaemia and swelling at day 7 postoperative is observed in 70% of infants with minimally retractile prepuces seen in infants circumcised before 1 year of age with subsequent bacterial carriage of skin commensals (70). Circumcised males have increased pain responses to childhood immunization 4–6 months post-surgery (71, 72) consistent with central sensitization (73). The abnormal development of sensory pathways in the developing nervous system elicited by the pain during critical postnatal periods is manifested in later life following nociceptive reexposure by abnormal sensory thresholds and pain responses that are not restricted to the original site of postnatal trauma (74–76). Neonatal nociceptive exposure induces long-term hypoalgesia or hyperalgesia depending on the nature and timing of the trauma (54, 77) and is consistent with surgery and pain adversely impacting neurodevelopment independent of anesthetic (76).

Post-circumcision, tactile hypersensitivity increases due to post-surgical and -traumatic mechanisms that contribute toward allostasis and the risk of SIDS. This is evident by the increase in toll-like receptor 4 (78) associated with post-circumcision wound healing, which is also observed in post-surgical tactile hypersensitivity in males and dependent on testosterone (79). Following peripheral nerve injury, the purinergic receptors in the spinal cord microglial cells release BDNF (79) and mitogen-activated protein kinase p38 (80) that contribute to neuropathic pain and tactile hypersensitivity. Due to their testosterone dependency, they are seen only in males (79). The testosterone surge occurring during the first 2- to 4-month period may increase susceptibility to the initial stages of infection and is consistent with the peak in SIDS mortality (81).

Male neonates subjected to circumcision can experience severe cardiorespiratory pain responses, including cyanosis, apnea, increased heart rate (82), and increased pitch (fundamental frequency) of cry (as high as 800–2000 Hz) associated with decreased heart rate variability, i.e., decreased vagotonia (83–85), a likely risk factor for SIDS. Other circumcision sequelae of sufficient severity to require emergency room evaluation or hospital admission and contribute toward allostasis include infection, urinary retention, inflammatory redness and swelling ascribed to healing (86, 87), and amputation/necrosis of the glans (88). Behavioral abnormalities, such as eating disturbance and disturbed sleep, are also the consequence of pain exposure (89).

Postoperative circumcision pain of ample severity to require analgesia is expected for about 10 days for healing with incomplete wound healing past day 14 seen in up to 6% of infants depends on the device used to amputate the foreskin (88), which is also associated with various adverse events (56, 90). The overall complication rate for circumcision ranges from 0.2 to 10% with many USA physicians performing the procedure without formal training, being unaware of contraindications, and incapable of handling post-op complications (56, 91, 92). Lower complication rates for early and late adverse events have been attributed to underreporting with late adverse events mistakenly not attributed to circumcision (92, 93). Consequently, the low number ascribed to circumcision as the cause of death (63) may be underreported and erroneously attributed to other causes, such as sepsis (94) or SIDS.

One mechanism by which circumcision may elicit SIDS concerns the inhibition of nerves involved in nociception processing that produces prolonged apnea while impairing cortical arousal. Neonatal surgery that traumatizes peripheral nerves with associated tactile hypersensitivity followed by a subsequent surgery later in development can increase spinal cord microglia signaling and elicit persistent hyperalgesia (80). It can also produce post-surgical hyperalgesia that subsequently alters postnatal development of the rostral rostroventral medulla (RVM), which controls the excitability of spinal neurons by spinally projecting neurons from the nucleus paragigantocellularis lateralis (PGCL) and the nucleus raphe magnus. Alterations in the RVM result in a descending inhibition of spinal reflex excitability on nociception (95). Inhibition of RVM neurons was shown to limit the duration of the laryngeal chemoreflex and produce prolonged apnea that contributes toward SIDS, particularly when combined with stimuli that inhibit respiration (96). In SIDS, norepinephrine, which depresses respiration, is increased in the PGCL and serotonin 5-HT_1A_ receptor that mediates nociceptive stimuli in the brainstem (97) and decreased in the raphe nuclei and the arcuate nuclei (98). The reduction in 5-HT_1A_ receptors observed in the brainstem of SIDS infants prompts the hypothesis that SIDS is caused by a brainstem abnormality that impairs the ability to generate protective responses to life-threatening challenges (99, 100). This hypothesis, however, does not explain why SIDS peaks at 2–4 months, rather than in an earlier GA (101). Orexin is another important regulator of both pain and sleep dysfunction. Orexin knockout mice presented greater degree of hyperalgesia induced by peripheral inflammation and less stress-induced analgesia than wild-type mice (102). In the rostral ventrolateral medulla and PGCL, orexin receptors are expressed in sympathoexcitatory bulbospinal neurons (103). A significantly decreased orexin immunoreactivity in the hypothalamus and pontine nuclei was observed in SIDS infants (104).

Another mechanism that can explain the SIDS toll following circumcision is the loss of ~1–2 ounces (oz) of blood out of a total of ~11 oz that a 3,000 gram male newborn has (105), the equivalent of ~1–2 blood donations in an adult. Excessive bleeding is highly common in circumcision with reports range from 0.1 to 35% (91, 106) in neonates. However, even moderate bleeding puts the infant as risk, and, being an inherent part of the procedure, it is not reported as a complication. Blood loss of 2–2.5 oz, ~15% of the total blood volume at birth, is sufficient to cause hypovolemia and death. Since a large fraction of newborns (26%), particularly premature infants, weigh much less than 3,000 grams (107), a smaller amount of blood loss may trigger hypovolemic shock. Therefore, when bleeding an infant of low birth weight or GA, the effect may be pathological resulting in a reduced blood pressure that has been associated with obstructive sleep apnea (OSA), a condition where the walls of the throat relax and narrow during sleep, interrupting normal breathing (108). It is, therefore, not surprising that most of the deaths following circumcision in high-income countries were due to bleeding (63). While it is accepted that failure of neural mechanisms causing arousal from sleep may play a role in at least some SIDS cases [e.g., Ref. (109)], it is unclear what causes the initial failure of the respiratory control (110). Comparing the breathing characteristics of 40 infants who eventually died of SIDS with 607 healthy controls, Kato and colleagues reported that SIDS infants have a greater proportion of obstructive and mixed apneic episodes than the control group (111). Although the frequency of these episodes decreased with age, the decrease was smaller in the SIDS infants than in the controls, in support of either immature or impaired respiratory control. Looking at the data by gender, however, shows that only boys exhibit a difference in apnea frequency in support of an impaired respiratory control (111), perhaps due to circumcision.

To date, circumcision in the USA, despite being the most common pediatric surgery, has not been subjected to the same systematic scientific scrutiny looking at immediate and delayed adverse effects, including pain [e.g., Ref. (112)], nor has circumcision status been included as part of a thorough SIDS investigation/registry or analyses [e.g., Ref. (2)] in spite of the male predominance of both neonatal circumcision and SIDS. However, based on assessment of risk of harms versus benefit, despite the latter including decreased risk of urinary tract infection (113), the Royal Australasian College of Physicians, the British Medical Association, the Canadian Paediatric Society (87), and several west European medical societies have recommended against routine neonatal circumcision (114), arguing that the benefits of circumcision to children are minimal, non-existent, or outweighed by the risks, and that circumcision is thereby unwarranted. The AAP’s recommendation in favor of this routine (115) has been widely criticized [e.g., Ref. (116)].

### Skin-Breaking Procedures

Skin-breaking procedures are a large and diverse group of insults that contribute toward allostasis. Infants subjected to repetitive heel lances, the most common skin-breaking procedure in neonates, have increased pain responses to subsequent skin cleansing and venipuncture (117). Heel lances in newborns elicit nociceptive-specific EEG brain activity associated with reflex withdrawal dependent on the stimulus intensity in the absence of clinical pain, which is difficult to measure (118). Brain imaging of newborn infants demonstrates increased sensitivity to nociceptive stimuli with greater amplitude and duration of reflex withdrawal compared to adults (119). Goksan and colleagues (2015) reported the activation of 18 out of 20 brain areas (including anterior cingulate cortex, bilateral thalamus, all divisions of insular cortex, and primary somatosensory cortex) in infants in response to nociceptive stimuli. Seven-day-old newborns do not have activation of the amygdala, orbitofrontal cortex, or anterior division of the insular cortex, suggesting that reward value/anticipation of future outcomes/emotional significance may not be attached to the nociceptive stimuli at this early postnatal stage of development, although activation of anterior cingulate cortex may suggest perceived unpleasantness (119). Non-specific neuronal bursting activity in response to heel lances is seen on EEG in preterm infants (<35 weeks of GA) while after 35–37 GA somatosensory potentials discriminate touch from nociceptive exposure (120). Exhibiting cortical activation following heel sticks is also associated with increase in heart rate and clinical pain behaviors (117), suggesting that preterm infants have at least the same increased pain response as neonates, with some studies suggesting a higher one (40).

The number of repetitive skin-breaking pain procedures (e.g., heel lance, intramuscular injection, tape removal, and intramuscular injection) in preterm infants (24–32 weeks) without neurodevelopmental, sensorimotor, or severe brain injury is significantly associated with decreased subcortical gray matter and maturation of white matter at term-equivalent age (40 weeks) (121). Overall, it is well established that intense and repetitive pain in early childhood is associated with negative life outcome (31) that contributes toward allostasis and thereby growing risk of SIDS.

### Inflammation, Birth Order, and Seasonality

Waning of maternal antibody levels and/or low levels of acquired immunity followed by recent infection and inflammation during a developmental period in the infant may result in a dysregulated inflammatory response, a risk factor for SIDS (122, 123). Approximately one-half of SIDS victims have a slight upper airway infection before death (124) with males reported to have an excess of infant mortality from respiratory deaths as well as SIDS (125). The risk of SIDS also increases with the number of siblings, otherwise known as “birth order” (124), another known SIDS risk factor. Mage and Donner (23) proposed that the higher risk of SIDS with increasing birth order could be due to greater probability of exposure to respiratory viral infection via contact with a sibling. This assumption is reasonable, but it does not explain the excess in male mortality.

The association between respiratory illnesses and seasonality is also elusive. In some countries, the winter seasonal predominance of SIDS has declined or disappeared when the prevalence of infants sleeping in the prone position has decreased, in support of an interaction between sleeping position and factors more common during colder months (e.g., overheating and infection) (19). However, in other countries, SIDS was shown to have a small seasonality component, suggesting that seasonality is a risk factor (23), though it also does not explain why males are at higher risk.

We speculate that the winter seasonal predominance of SIDS is due to the increase in respiratory illnesses among household members that are in contact with the infant (126), particularly older siblings, that contributes toward the infant’s allostatic load. Infants lose the protection of maternally acquired antibodies at 2–4 months of age (127), when they become susceptible to upper airway infections. At that time, even otherwise benign upper respiratory infections can augment the laryngeal cough reflex and produce prolonged apnea, an important risk factor for SIDS. This risk may be elevated among infants with an impaired immune system (128) and further elevated among circumcised infants struggling to regulate their allostatic exposures to the recent and new stressors (26) (Figure 1).

### The Nightly SIDS Cascade

Compared with other infants, those who subsequently succumb to SIDS have higher heart rates, reduced heart rate variations, abnormal QT intervals, increased baseline heart rates, and bradycardias preceding apnea or during ventilatory effort (129). In this section, we highlight some of the critical events in the nightly SIDS cascade.

Depending on the GA, nearly 90% of preterm infants experience intermittent hypoxia or recurrent apneas (130) attributable to central apnea, with obstructed breaths appearing during periods of prolonged central apnea (131). OSAs have been associated with postoperative pain (132, 133), increased norepinephrine and epinephrine levels (134), higher blood pressure (135), and upper airway hypotonia during rapid eye movement (REM) sleep due to decreased genioglossus activity (136). Repetitive apneas have also been associated with decreased arterial oxygen saturation and decreased cerebral oxygenation, which would have gone undiagnosed and thereby contribute toward the SIDS diagnosis (101). Such repetitive apneas may also contribute to higher cerebral oxidative stress, increased sympathetic activity, and hypoxic loss of neurons that alter or become less efficient in adulthood (137). These events are more frequent in the supine position and when the tonsils are swollen which accompany a number of upper respiratory tract infections. Unsurprisingly, infants succumbing to SIDS, who have been monitored, experienced significantly more frequent episodes of obstructive and mixed sleep apnea, especially males (111) during REM sleep (138). SIDS infants experience fewer cortical arousals during REM and non-REM (NREM) sleep, more subcortical activations in REM sleep of longer durations in both REM and NREM sleep, more frequent subcortical activations in the first part of night, and fewer cortical arousals in the early AM hours (138). Polygraphic changes, considered pathological, recorded in the brainstem of SIDS infants include apoptosis, hypoplasia, and gliosis (138).

Unusual breathing patterns and decreased heart rate variability may thereby indicate at-risk infants. Breathing patterns influence cardiovascular response (131, 139) with decreased heart rate variability in the neonate attributable in part to increased sympathetic activity and/or decreased parasympathetic activity with gradually increasing parasympathetic activity over the first year of life (140–142). Infants normally spend about 70% of a 24 h period asleep, and those who subsequently succumb to SIDS demonstrate a decreased heart rate variability due to increased sympathetic activity and/or decreased parasympathetic activity in all sleep phases (active/quiet sleep) (129, 140). Sympathetic activation/catecholamine release in response to hypoxia/hypercapnia is mediated by carotid body glomus cells acutely (143) and by peripheral chemoreceptor organs [e.g., adrenal medulla and oxygen sensing pulmonary neuroendocrine cells (PNEC)] (144). Lungs from infants dying of SIDS have demonstrated hyperplasia of PNEC with reduced myelination of its vagal afferents impairing oxygen sensing (145) as well as increased airway (146) and thick pulmonary arteries in males (147).

Channelopathies can induce cardiac arrhythmias, such as abnormal QT syndromes, catecholaminergic ventricular tachycardia, and prolonged QT interval associated with intracellular acidosis (148). We note that SIDS diagnosis may not be given to infants that suffer lethal arrhythmias or diagnosed with channelopathies, however channelopathies can inflict sudden unexpected death during infancy via a lethal ventricular arrhythmia without leaving a trace of structural evidence detectable during postmortem examination, in which case they are candidate suspects in the etiology of SIDS (149). Increasing QT interval is noted during early development up to four postnatal months followed by decreasing ECG’s QT and PR intervals during childhood (150). Prolonged QT has been long associated with SIDS (151) and is a risk factor for ventricular arryhthmia with 50% of infants succumbing to SIDS observed to have prolonged QT interval during the first week of life (150). Hypoxia interferes with hemichannel function and maturation of the cardiac conduction system, which increases the risk of arrhythmogenic death (150). In this respect, mutations in genes associated with development of the cardiac conduction system (152) and cardiomyopathy (153) have been implied in SIDS, although the extent to which they contribute to SIDS remains under debate. While reports have estimated that up to 15% of SIDS were related to specific genetic variants [e.g., Ref. (154)], a recent study that sequenced the full exons of 64 genes associated with sudden death in the largest known cohort (351) of infant and young sudden death decedents reported that less than 4% of unexpected deaths were associated with a pathogenic genetic variant (155). These results suggest that many pathogenic variants involved in SIDS are unknown or, most likely, that pathogenic variants play a minute role is SIDS.

### The Significance of the Allostatic Load Model for SIDS

Sudden infant death syndrome occurs when an infant dies suddenly, unexpectedly, and without a cause identified through a forensic autopsy or death-scene investigation. We speculate that SIDS is caused by prolonged and repetitive iatrogenic stressful, painful, or traumatic experiences during critical development stages that constitute allostatic overload (156). Over the past years, allostatic load models were proposed to explain several leading medical conditions, including mental health disorders (157, 158), preterm birth (159), and chronic stress (160).

While the infant’s first environment is typically romanticized as peaceful, painless, hygienic, safe, and harmless, in practicality it may be anything but that. Already in the uterus, the fetus may be exposed to maternal substance use (e.g., smoking and drug use) associated with SIDS (19, 161). During a prolonged hospitalization in the Neonatal Intensive Care Unit that follows a preterm birth, infants may be exposed to extended and repeated pain, which thier unstable and immature physiological systems are unable to offset and will potentially render them more vulnerable to the effects of repeated invasive procedures (38). Neonatal circumcision typically involves maternal separation, pain, bleeding, and shock and, like any operation, puts the infant at risks of hemorrhage and sepsis even when anesthetic is used (67). The long-term consequences of circumcision include, among else, greater pain response to routine immunizations within the few months past birth (72). During winter time, the infant is at risk of infection and illnesses that grows with the number of household members, particularly older children (126), which explains why an elevated immune response is one of the hallmarks of SIDS (123, 128). Other common stressors may include birth trauma, birth injury, traumatic injury, life-threatening event, inadequate nutrition, heel lances, prolonged institutionalization, skin breaks, and air pollution – all contribute to the build-up of toxic allostatic load.

Our model represents a major departure from previous models, such as the “three interrelated causal spheres of influence model” that requires two out of three factors to act simultaneously (subclinical tissue damage, deficiency in postnatal development of reflexes and responses, and environmental factors) (162), or the more popular “triple-risk model,” which advocates that the combined effect of three factors (vulnerable infant, critical development period, and environmental stressors) causes SIDS (163). Our model posits that any infant may succumb to SIDS when the combined and cumulative effect of the environmental stressors has exceeded their tolerance level shaped by their unique genetic and environmental factors (Figure 1).

## Testing the Hypothesis

Our hypothesis makes several testable predictions.

### Neonatal Circumcision is a Risk Factor for SIDS

Double-blinded case–control human studies aiming to test our hypothesis are unfeasible due to ethical consideration and the difficulties in matching cases and controls (19). Fortunately, the prepuce has been well conserved throughout mammalian evolution (164), which attests to its functional importance, and allows carrying out animal studies. Our hypothesis can be tested by circumcising the prepuce of mammalian animal models and measuring whether an excess of SIDS is observed among cases when compared with untreated controls. Curiously, none of the studies purporting the “benefits” of neonatal circumcision has ever been demonstrated using animal models, which are the only viable means to carry out double-blinded case–control studies assessing the short- and long-term health impacts of circumcision. In humans, we can expect higher SIDS rates in Anglophone countries that adopted male neonatal circumcision in the nineteenth century, compared to Iberio-American that traditionally have opposed circumcision (66). We can also expect a higher incidence of SIDS in USA states where Medicaid, the most common health insurance, covers circumcision, compares to states where this procedure is not covered by Medicaid after accounting for culture and socioeconomic status. The data for such study can be obtained from the CDC’s SIDS registry (165). Finally, we can compare the circumcision status of SIDS victims versus healthy controls, obtained through autopsies and questionnaires, respectively. New genetic tools, such as Case-control matcher (http://www.elhaik-lab.group.shef.ac.uk/ElhaikLab/index.php), based on biogeographic ancestry tools [e.g., Ref. (166)], can be instrumental in optimizing case–control matches by identifying individuals that have similar population structure and genetic background and minimizing the bias studies due to population stratification.

### Male Neonatal Circumcision Accounts for a Large Fraction of the Gender Bias in SIDS

We speculate that the male bias in SIDS observed in western countries may be due to both natural protections that render females more resilient to nociceptive stimuli and legal-cultural ones that protect females from circumcision in these countries. The weights of these two factors are unknown, yet we expect the gender deviations from even proportions in SIDS to be correlated with circumcision rates. Consequently, large male bias is expected in societies that practice neonatal circumcision whereas smaller bias is expected in societies that circumcise both males and females or avoid it altogether.

### Circumcised Premature Infants Are at High Risk

We predict that circumcised premature infants would be at higher risk for SIDS compared with intact preterm infants. This can be tested by an analysis of hospital records after properly matching cases with controls (19).

Additional complications that should be considered when testing these predictions in humans include misclassification of SIDS to other categories, inconsistent reports of SIDS over time in certain countries due to changes in definitions, inconsistent reports of circumcision (167), and the absence of legislation requiring an autopsy or thorough death-scene investigation.

## Implications of the Hypothesis

Our hypothesis denotes that while some infants are genetically more vulnerable to the effects of allostatic load, all infants living in a stress fraught environment may be at risk of SIDS. If proven, this hypothesis will generate a paradigm shift in our understanding of neonates and pain toward focusing on factors that contribute toward allostasis.

The implication of our hypothesis, which explains many of the findings already reported in the literature, is that environmental risks should be mapped and eliminated or mitigated to reduce cases of infant deaths. Many of these implications can be put to use immediately, such as applying pain management techniques to infants that experience repetitive pain, eliminating neonatal circumcisions when possible, and postponing non-medical circumcisions to later ages. We note that postponing the circumcision does not alleviate individuals from associated complications. Studies of female circumcision among adolescents consistently report major physical, psychological, and sociological complications following the surgery (168, 169).

Although at the time being identifying infants at higher risk for SIDS is impossible, our hypothesis predicts that they would be highly sensitive to pain which can be estimated, for example, from the pitch and tone of their cry following negative stimulus (170). Such approach requires developing a standardized method that yields a small percentage of false positives. Progress should also be made in screening for genetic variants that increase the risk for SIDS.

In summary, SIDS is a complex, multifactorial syndrome in which continued research is needed to fully understand the relevant interactions between genetic and environmental risk factors that affect causation. We introduced an allostatic load model to explain SIDS and argued that it explains the main characteristic of this syndrome (Table 1). We also proposed how to test the hypothesis and offered guidelines on how to reduce the risk of SIDS.

## Author Contributions

EE developed the hypothesis and wrote the paper.

## Conflict of Interest Statement

The author declares that the research was conducted in the absence of any commercial or financial relationships that could be construed as a potential conflict of interest.
